# Molecular mechanisms of air pollution–induced carcinogenesis and the emerging role of microplastics

**DOI:** 10.1186/s40246-025-00880-0

**Published:** 2025-12-04

**Authors:** Julia Vu, Kari Nadeau, Maya Kasowski

**Affiliations:** 1 Department of Bioengineering, Stanford University, 240 Pasteur Dr., Palo Alto, CA 94304 USA; 2 Department of Environmental Health, Harvard University, Cambridge, USA; 3 Sean N. Parker Center for Allergy and Asthma Research, Stanford University, Stanford, USA; 4 Dept. of Pathology, Stanford University School of Medicine, Stanford, USA

**Keywords:** Air pollution, Particulate matter, Carcinogenesis, Microplastics, Environmental exposome

## Abstract

Ambient air pollution is a major environmental carcinogen consisting of a complex mixture of particulate matter, gases, and adsorbed toxicants. Fine (PM_2.5_) and ultrafine (PM_0.1_) particles are of particular concern due to their capacity to penetrate deep into the lungs and translocate systemically, carrying carcinogens such as heavy metals, volatile organic compounds (VOCs), and polycyclic aromatic hydrocarbons (PAHs). Exposure arises from traffic emissions, industrial activity, biomass combustion, and indoor sources, with inhalation as the primary route. Epidemiologic studies have firmly established associations between air pollution, particularly PM_2.5_ and increased risks of lung, bladder, breast, and hematologic cancers, even at concentrations below regulatory thresholds. Mechanistically, pollutant-induced carcinogenesis is driven by oxidative stress, DNA damage, epigenetic reprogramming, immune dysregulation, and impaired cell cycle control. Air pollution generates reactive oxygen species (ROS), disrupts mitochondrial function, alters DNA repair pathways, and modulates the expression of tumor suppressor genes through methylation and histone modifications. Prolonged inflammation and immune suppression in polluted tissue microenvironments further promote malignant transformation. Recent studies have shown increased interest in microplastics (MPs) as potential environmental carcinogens, given their unique physical properties and poorly characterized toxicological profiles. Preliminary findings indicate that microplastics are detectable in multiple cancer types and may correlate with distinct molecular alterations, suggesting a link to carcinogenesis and highlighting a critical future direction for environmental cancer research.

**Graphical abstract:**

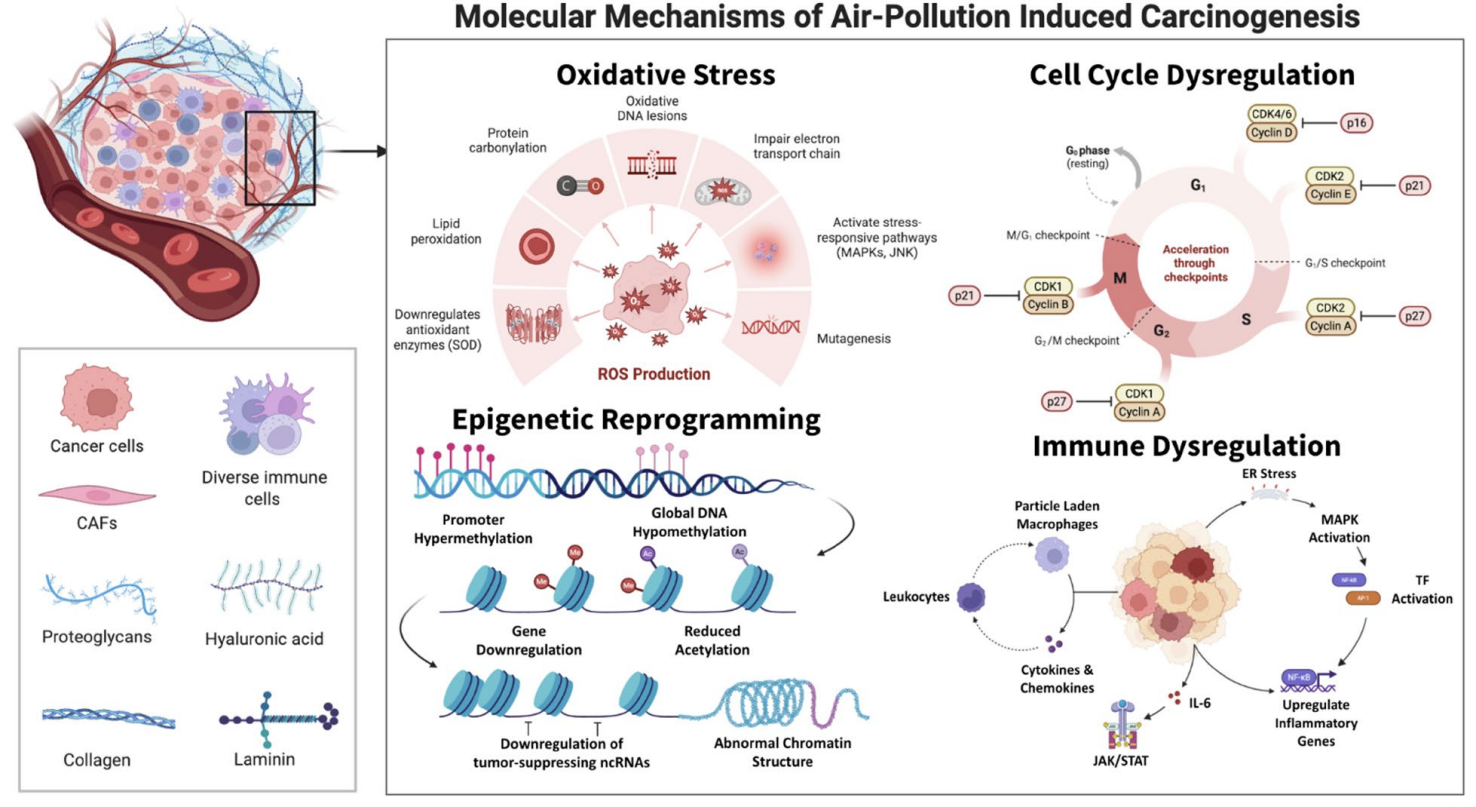

## Introduction

While the focus of human cancer research has traditionally centered on genomics, there is increasing recognition of the influence of environmental exposures and the broader exposome on cancer initiation, progression, and interindividual risk variability [1, 2]. Air pollution is increasingly recognized as a leading modifiable cause of cancer worldwide. In 2013, the IARC designated outdoor air pollution as a Group 1 carcinogen, based on consistent links to lung, breast, and several other tumors [3]. Globally, millions of premature deaths per year (primarily cardiovascular and respiratory) are attributed to polluted air, and epidemiological data rank air pollution as a top contributor to lung, bladder, and other cancers [4–7]. Rapid industrialization and urbanization have increased population exposures to complex pollutant mixtures, including fine particles, combustion byproducts, and chemicals from traffic, industry, and indoor sources. These exposures impact fundamental biological pathways known to drive carcinogenesis, including oxidative damage, DNA mutations, chronic inflammation, and epigenetic disruption [8–11]. The purpose of this narrative review is to synthesize current knowledge on the molecular mechanisms by which air pollution drives cancer and highlight microplastics as an emerging and understudied contributor to environmental carcinogenesis.

## Air pollution exposome

### Major carcinogenic components

Ambient air pollution is a heterogeneous mixture of particles and gases. Fine particulate matter (PM) is of particular concern because its small aerodynamic diameter determines how deeply it can penetrate the respiratory tract: coarse particles (PM_10_, ≤ 10 μm) are typically deposited in the upper airways, whereas fine particles (PM_2.5_, ≤ 2.5 μm) can reach the bronchioles and alveolar regions of the lungs [12] (Table 1). These ultrafine particles (< 0.1 μm) can translocate into circulation, carrying adsorbed toxins, including transition metals (lead, cadmium, nickel, and mercury), organic compounds (polycyclic aromatic hydrocarbons, like benzo[a]pyrene), volatile organic compounds (VOCs, like benzene and formaldehyde), and combustion residues (soot and black carbon) [13, 14]. Major carcinogenic constituents of particulate matter include sulfates, organic compounds, polycyclic aromatic hydrocarbons (PAHs), and heavy metals such as arsenic, cadmium, and lead [15, 16]. A principal anthropogenic source of PM_2.5_ is diesel exhaust, which contains high levels of PAHs and nitro-PAHs and is classified as a Group 1 human carcinogen by the IARC [17–19]. Traffic-related air pollution (TRAP) is a significant source of PM and mutagens implicated as carcinogens in urban areas [19, 20].

**Table 1:** Major air pollutants linked to cancer and associated mechanistic effects [3, 21]

Pollutant	Examples/sources	Mechanistic effects	Cancer types associated
PM_2.5_ (fine PM)	Traffic exhaust (diesel/gasoline), coal power, biomass burning, wildfire smoke	High surface area carriers of adsorbed toxins; contains metals (Fe, Cu), PAHs, organics [22–24]. Generates ROS; induces inflammation; DNA adducts via attached PAHs; epigenetic changes.	Lung, bladder, breast, cardiovascular outcomes [25–28]
Ultrafine particles (PM_0.1_)	Vehicle emissions, industrial emissions	Penetrate cell membranes and blood; rich in organic carbon and metals. Induce oxidative stress and systemic inflammation [29]	Similar to PM_2.5_, inc. neurological effects, cancer in multiple organs [13]
Diesel exhaust	Diesel engines (trucks, generators)	High PAH and nitro-PAH load; persistent organic carbon. Potent ROS generator; aryl hydrocarbon receptor (AhR) activation; inflammation via IL-6, TNF-α [30, 31]	Lung (adenocarcinoma), bladder, skin (squamous), and more [32]
Polycyclic aromatic hydrocarbons (PAHs)	Incomplete combustion (vehicle, cooking smoke, wood burning)	Form DNA adducts (e.g. BPDE from benzo[a]pyrene); cause bulky DNA lesions and error-prone repair [33]. Also induce ROS and chronic toxicity.	Lung, skin, bladder, hematopoietic cancers [34]
Volatile organic compounds (VOCs)	Industrial solvents, gasoline fumes, incense smoke	Benzene: hematotoxic and carcinogenic (leukemia); formaldehyde: DNA crosslinks, epigenetic effects [35, 36]. Induce oxidative and genotoxic stress in exposed cells.	Leukemia (benzene), nasal/pharyngeal cancers (formaldehyde) [37]
Heavy metals (As, Cd, Ni, etc.)	Mining, smelting, power plants, battery factories	Generate Fenton chemistry (ROS); inhibit DNA repair; replace essential metal cofactors; induce epigenetic aberrations [38].	Skin, lung (As, Ni), bladder (As), prostate (Cd) [39]

### Sources and exposure routes

Air pollution exposures vary by setting and individual activity. Ambient air pollution arises from a complex mixture of sources, including vehicular emissions (e.g., tailpipe exhaust, brake and tire abrasion, resuspended road dust), industrial activities (such as combustion processes, smelting, and manufacturing), power generation (especially coal-fired plants), and natural phenomena such as wildfires and dust storms. Urban environments are often characterized by elevated levels of fine particulate matter (PM_2.5_), nitrogen oxides (NO_X_), and ground-level ozone (O₃), driven by high traffic density and industrial output [40]. In contrast, rural areas in low- and middle-income countries frequently experience substantial air pollution from indoor and community-level biomass burning, coal-based heating, and agricultural practices [41].

Inhalation is the predominant exposure route. Fine particles (PM_2.5_) penetrate deep into the bronchioles and alveoli, whereas ultrafine particles (PM_0.1_) and volatile gases are capable of translocating across alveolar-capillary barriers into the bloodstream [12, 13]. Indoor exposures (cooking and heating with biomass fuels, tobacco smoke) can produce PM_2.5_ and VOC levels orders of magnitude above outdoor levels, especially without ventilation [42]. Individuals in transportation, mining, manufacturing, and construction frequently encounter pollutant levels substantially above ambient averages. Traffic police officers, truck drivers, toll booth workers, and road maintenance personnel exhibit elevated risks of lung cancer, largely attributable to chronic inhalation of diesel exhaust, PM_2.5_, and associated carcinogens such as polycyclic aromatic hydrocarbons [43, 44]. Ultimately, an individual’s lifetime exposure (“cumulative dose”) depends on geographical location, occupation, socioeconomic status, and personal behaviors.

### Classification and epidemiologic links to cancer

The carcinogenicity of air pollution is well-documented. Based on evaluations conducted between 2010 and 2016, the International Agency for Research on Cancer (IARC) classifies both outdoor air pollution and diesel engine exhaust as carcinogens [45]. Epidemiologic studies consistently demonstrate a robust association between fine particulate matter (PM_2.5_) exposure and increased lung cancer risk, even at concentrations below current regulatory thresholds [26]. Meta-analyses estimate that each 10 µg/m^3^ increase in PM_2.5_ corresponds to an approximately 10–15% elevation in lung cancer incidence [26]. Large-scale prospective cohorts corroborate these findings across diverse populations and geographies [46, 47]. Moreover, data suggest dose-dependent associations between air pollution and other malignancies, including breast, bladder, hematologic cancers, and pediatric leukemias, though these relationships remain less well-characterized [48]. Indoor exposures, particularly from coal and biomass combustion, have been strongly linked to adenocarcinoma of the lung and upper respiratory tract cancers, especially in non-smoking populations [49].

Over half of global pollution-attributable deaths occur in Asia and Africa, where exposure levels are often highest and access to clean fuels is limited [50]. Women who cook with biomass fuels in poorly ventilated homes face substantially elevated risks of lung cancer despite having no history of smoking [51]. Low-income and minority communities are disproportionately situated near major roads, industrial facilities, and other high-emission zones, exacerbating their cancer burden [52].

## Molecular mechanisms of air pollution-induced carcinogenesis

Airborne carcinogens trigger several interrelated molecular pathways. Inhaled particles and chemicals generate reactive oxygen species (ROS), directly damage DNA and organelles, and hijack signaling networks that control inflammation, cell cycle, and gene expression. Repeated or chronic activation of these pathways leads to mutations, epigenetic reprogramming, and an environment conducive to malignant transformation.

### Oxidative stress and ROS generation

Airborne particulate pollutants and their associated chemical constituents are potent inducers of oxidative stress, primarily through the generation of reactive oxygen species (ROS) such as superoxide anions (·O_2_^−^), hydroxyl radicals (·OH), and hydrogen peroxide (H_2_O_2_) [53, 54]. Transition metals commonly found on particle surfaces, such as copper (Cu), iron (Fe), and manganese (Mn), are particularly critical mediators of oxidative injury by catalyzing Fenton and Fenton-like reactions, in which ferrous ions (Fe^2+^) react with hydrogen peroxide to yield hydroxyl radicals (·OH) [55–57]. These reactions are autocatalytic and self-propagating, as the oxidized metal ions (Fe^3+^, Cu^2+^) are readily reduced back to their active states by cellular reductants, thereby sustaining radical flux [56].

Concurrently, organic pollutants, including quinones and polycyclic aromatic hydrocarbons (PAHs), contribute to ROS production via enzymatic and non-enzymatic redox cycling mechanisms [58]. Metabolic activation by cytochrome P450 enzymes and NADPH–cytochrome P450 reductase produces semiquinone radicals that continuously shuttle electrons to molecular oxygen, generating superoxide (O_2_^−^) and hydrogen peroxide (H_2_O_2_) [59]. Fine particulate matter (PM_2.5_) exhibits especially high oxidative potential due to its enrichment in redox-active metals and secondary organic aerosols [60]. In vitro PM_2.5_ exposure in cultured pulmonary and cardiovascular cells significantly elevates intracellular ROS levels, depletes endogenous antioxidant defenses, and downregulates critical antioxidant enzymes such as superoxide dismutase (SOD) and catalase [22–24, 61]. The resulting secondary ROS generation leads to widespread macromolecular damage, including lipid peroxidation, protein carbonylation, and oxidative DNA lesions [62, 63]. Lipid peroxidation products such as 4-hydroxynonenal (4-HNE) and malondialdehyde (MDA) accumulate following exposure to air pollution, forming adducts that disrupt membrane integrity and signaling proteins [64, 65].

Mitochondria are particularly susceptible to pollutant-induced oxidative stress. PM_2.5_ disrupts mitochondrial membrane potential, induces calcium efflux, impairs complex I and III of the electron transport chain, and releases cytochrome c into the cytosol, thereby amplifying ROS production and reducing ATP synthesis [23, 63]. These mitochondrial perturbations not only exacerbate cellular redox imbalance but also serve as upstream triggers for redox-sensitive signaling cascades.

These redox disturbances propagate through intracellular signaling networks that trigger transcriptional and inflammatory responses in response to oxidative cues. Persistent oxidative stress activates several stress-responsive pathways, including mitogen-activated protein kinases (MAPKs), including ERK1/2, JNK, and p38, which converge on redox-sensitive transcription factors such as nuclear factor κB (NF-κB), activator protein 1 (AP-1), and nuclear factor erythroid 2–related factor 2 (Nrf2) [66, 67]. Nrf2 activation causes the transcription factor to dissociate from its cytoplasmic repressor Keap1 and translocates to the nucleus, where it upregulates antioxidant and detoxification genes, like HO-1, NQO1, GCLM [68–70]. On the other hand, NF-κB and AP-1 activation upregulates pro-inflammatory and pro-survival gene expression, including IL6, IL8, TNFA, and COX2, leading to a cytokine-rich microenvironment and chronic inflammation [68, 69, 71]. In vitro nontoxic PM_2.5_ exposure to pulmonary epithelial and cardiac tissue reveal that PM2.5 exposure induces endoplasmic reticulum (ER) stress and calcium efflux, leading to phosphorylation of MAPKs and degradation of IκB, the inhibitory subunit that normally sequesters NF-κB in the cytoplasm [72–74]. This cascade culminates in nuclear accumulation of NF-κB (p65) and AP-1 (c-Fos/c-Jun), initiating transcription of inflammatory mediators that sustain oxidative feedback loops [75]. The resultant secretion of interleukin-6 (IL-6), interleukin-8 (IL-8), and tumor necrosis factor alpha (TNF-α) recruits immune cells and perpetuates chronic inflammation [76].

When antioxidant buffering capacity is overwhelmed due to high pollutant burden or host susceptibility factors such as GSTM1 gene deletion, cells accumulate oxidative damage. Oxidative DNA lesions, including 8-hydroxy-2’-deoxyguanosine (8-oxo-dG), are also significantly associated with ambient PM_2.5_ exposure, indicating oxidative DNA damage, specifically a G → T transversion mutation, and redox imbalance in urine and nasal epithelial cells of individuals exposed to air pollution [67, 77, 78]. Exposure of pulmonary epithelial cells in vitro to non-toxic levels of PM2.5 resulted in an elevation of 5′-bromodeoxyuridine, a marker of unscheduled DNA synthesis and/or cell proliferation, indicating pollutant-induced activation of DNA repair and replicative stress responses [75]. Unrepaired oxidative lesions contribute to mutagenesis via miscoding and replication errors, laying the groundwork for genomic instability. Airborne pollutants, notably PM_2.5_, shift the cellular redox balance toward a sustained pro-oxidant state. This oxidative stress is a central mechanism by which air pollution promotes DNA damage, disrupts signaling pathways, and initiates the molecular cascade leading to carcinogenesis.

### DNA Damage, Genotoxicity, and mutagenesis

Air pollution contributes to carcinogenesis through the induction of direct and indirect DNA damage, leading to genomic instability and mutagenesis. Among the most potent agents are lipophilic organics within fine particulate matter (PM2.5), particularly polycyclic aromatic hydrocarbons (PAHs) such as benzo[a]pyrene (BaP). Once inhaled, PAHs undergo metabolic activation via cytochrome P450 enzymes (primarily CYP1A1 and CYP1B1) under the control of the aryl hydrocarbon receptor (AhR) [79, 80].

PAHs activation produces reactive diol epoxides, like benzo[a]pyrene diol epoxide (BPDE), that covalently bind to nucleophilic sites on DNA bases, especially the N2 position of guanine [33]. These bulky DNA adducts distort the double helix, impede polymerase progression, and recruit the nucleotide excision repair (NER) machinery [81]. When repair is incomplete or erroneous, replication bypass across these adducts introduces G→T and G→A transversions, hallmark mutations observed in smoking- and pollution-associated lung cancers [82]. Elevated levels of PAH–DNA adducts have been documented in airway epithelial cells following exposure to ambient air pollution and in peripheral blood lymphocytes of occupationally exposed individuals [83–86].

Concurrently, the redox-active metals and organic radicals in PM_2.5_ drive the production of ROS that inflict oxidative DNA damage. Hydroxyl radicals (·OH) and superoxide anions (O_2_^–^) attack the deoxyribose backbone and bases, forming oxidized lesions such as 8-hydroxy-2′-deoxyguanosine (8-oxo-dG), abasic (AP) sites, and both single- and double-strand breaks [87, 88]. These lesions overwhelm base excision repair (BER) pathways and activate poly(ADP-ribose) polymerase (PARP)-mediated responses that, when excessive, can lead to energy depletion and reduced cell viability [89]. In vitro studies using human bronchial epithelial cells and in vivo murine models have consistently demonstrated that PM2.5 exposure increases DNA strand breaks, chromosomal aberrations, and micronuclei formation through combined inflammatory and oxidative mechanisms [90, 91]. Ambient levels of diesel exhaust particles and PM exposure have been shown to induce chromosomal aberrations and DSBs in human bronchial epithelial cells and murine lung tissue [92].

Beyond inducing DNA damage, air pollution also compromises the cell’s endogenous DNA repair systems. Chronic PM exposure has been shown to downregulate DNA repair capacity at both transcriptional and epigenetic levels. Persistent PM_2.5_ exposure has been associated with promoter hypermethylation and transcriptional silencing of key genes involved in homologous recombination (HR), such as *RAD51*, thereby diminishing the cell’s ability to accurately repair DSBs [93, 94]. Similarly, aberrant methylation and reduced expression of BER and NER genes, including OGG1, MGMT, and XPA, have been observed in exposed epithelial cells and peripheral leukocytes, facilitating accumulation of unrepaired lesions and mutagenesis [95–97]. The repression of OGG1, which normally excises 8-oxo-dG, allows oxidized guanine lesions to persist through replication and modulating transcription of genes involved in various cellular processes [98]. MGMT silencing prevents the removal of mutagenic O^6^-methylguanine adducts, predisposing cells to G→A transitions in oncogenes and tumor suppressors such as TP53 and KRAS [99].

Epidemiological evidence supports this mechanistic framework. Whole-exome analyses of lung tumors from heavily polluted regions of China revealed an enrichment of somatic mutation burden, with an average of 68 nonsynonymous mutations per tumor, compared to approximately 22 in control-region cancers, implicating chronic exposure to pollutants in genomic instability [100]. Rodents subjected to chronic urban PM exposure developed lung adenocarcinomas at a higher frequency and exhibit genomic instability across multiple tissues, including the lungs and liver [101, 102]. Human biomonitoring studies in high-exposure cohorts consistently reveal elevated biomarkers of genotoxic stress, including increased micronucleus formation, longer comet assay tail lengths, and higher systemic levels of 8-oxo-dG [103]. Inhaled pollutants initiate DNA adduct formation and oxidative injury, subvert DNA repair pathways through epigenetic repression, and ultimately increase the somatic mutation burden that contributes to carcinogenesis.

### Epigenetic reprogramming

In addition to its mutagenic effects on DNA sequence, air pollutants exert profound effects on the epigenome, including changes in DNA methylation, histone modifications, and non-coding RNA expression, that can persist long after the initial exposure and contribute to long-term shifts in cellular phenotype [104]. Multiple studies have shown that inhaled particulate matter (particularly PM_2.5_) and associated chemical toxicants modulate DNA methylation patterns in both target tissues and peripheral blood [105]. Short-term PM_2.5_ exposure has been associated with acute hypomethylation of repetitive elements such as LINE-1 and site-specific methylation changes in tumor suppressor genes in circulating leukocytes [48, 106–108]. Chronic exposure, particularly in urban or industrial settings, leads to a characteristic pattern of global DNA hypomethylation, contributing to genomic instability, and promoter-specific hypermethylation, which can silence genes critical for tumor suppression [109, 110]. Repetitive genomic elements such as LINE-1 and Alu sequences often become hypomethylated, leading to chromosomal instability and inappropriate activation of transposable elements [111]. Conversely, CpG islands within the promoters of tumor suppressor genes, including TP53, CDKN2A (p16), and RASSF1A, undergo hypermethylation, resulting in their transcriptional silencing, following low-dose PM_2.5_ exposure in alveolar epithelial cells [112]. Prolonged PM_2.5_ exposure in bronchial epithelial cells drives DNMT3B recruitment to the TP53 promoter, silencing p53 expression and disabling key checkpoint responses to DNA damage [112].

PM-induced epigenetic repression can downregulate DNA repair machinery. PM_2.5_ can drive hypermethylation of tumor-suppressive and repair genes such as RAD51, SOX2, and DLEC1, and hypomethylation of proto-oncogenic or stress-response genes including AHRR, OGG1, and NAT10 [113–116]. Hypermethylation of RAD51, a critical enzyme for homologous recombination and double-strand break repair, compromises genomic stability and increases mutagenesis [94]. SOX2, typically an oncogenic transcription factor in lung and esophageal cancers, is significantly hypermethylated in individuals residing in areas with higher industrial air pollution [117]. DLEC1, a well-recognized tumor suppressor and biomarker, undergoes promoter hypermethylation under PM_2.5_ exposure, resulting in transcriptional silencing that favors cell cycle progression and reduced apoptosis [118]. Interestingly, physical exercise has been shown to mitigate DLEC1 hypermethylation, suggesting a possible lifestyle-based epigenetic intervention [118, 119]. Downregulation of DNA repair machinery diminish the cell’s capacity to correct oxidative lesions and adducts, facilitating the fixation of mutations. Importantly, these pollutant-induced epigenetic marks persist across cell divisions, conferring heritable alterations that predispose epithelial progenitors to transformation [120].

Air pollution also reconfigures chromatin structure through shifts in histone acetylation and methylation dynamics. Oxidative stress generated by pollutants has been shown to disrupt the activity of histone-modifying enzymes, including histone acetyltransferases (HATs) and deacetylases (HDACs) [121]. ROS-mediated inactivation of HDACs and enhanced HAT activity increase histone H3/H4 acetylation, particularly at inflammatory gene promoters, resulting in persistent histone acetylation marks (e.g., H3K9ac, H4K16ac) that sustain transcription of pro-inflammatory mediators [122]. PM_2.5_ exposure increases histone H4 acetylation at the IL8 promoter, maintaining its transcriptional activation and reinforcing local inflammation [123]. Similar pollutant-induced histone modifications at COX2 and MMP9 loci promote prostaglandin synthesis and matrix remodeling, contributing to tumor invasion and metastasis [124]. PM-driven increases in H3K4me3 and H3K27ac globally activate pathways involved in cell survival and metabolic reprogramming [125].

Non-coding RNAs (ncRNAs), including microRNAs (miRNAs) and long non-coding RNAs (lncRNAs), are also dynamically regulated by air pollutant exposure. Chronic PM_2.5_ exposure alters the expression of miRNAs that regulate oncogenic and tumor-suppressive pathways [126]. Tumor-suppressive miRNAs such as miR-185, miR-182, and miR-126 are consistently downregulated upon exposure, repressing targets that promote proliferation (BCL2, EGFR, PIK3CA) and survival [126]. miR-17, miR-18a, and miR-20a, components of the miR-17/92 oncogenic cluster, are downregulated upon PM_2.5_ exposure, repressing the tumor-suppressive 15-LOX2 gene and promoting uncontrolled proliferation [127]. Conversely, pro-inflammatory miRNAs like miR-146a and miR-21 are upregulated via NF-κB and STAT3 activation, amplifying immune dysregulation [128, 129]. miR-200 family members (e.g., miR-200a-3p) are similarly elevated, enhancing cell migration and epithelial–mesenchymal transition (EMT) by downregulating TNS3 [130]. miR-582-3p is upregulated by ambient PM_2.5_ exposure and activates the Wnt/β-catenin pathway, further driving EMT and invasiveness [131].

Recent work has identified oxidative stress-induced lncRNAs, such as LOC146880, which is upregulated following PM2.5 exposure and promotes autophagy and malignant transformation in bronchial epithelial cells through regulation of the mTOR pathway [132, 133]. Concurrently, pollutant exposure increases expression of the lncRNA NEAT1, along with cancer stem cell markers CD133 and CD44, promoting tumor growth and metastasis [134]. miR-582-5p directly targets NEAT1 to suppress these oncogenic effects, suggesting a regulatory feedback mechanism that may be disrupted by chronic exposure [134]. These RNA-based regulators reinforce the pollutant-induced transcriptional changes and establish a long-lasting “epigenetic memory” that allows for persistence of the altered state even in the absence of continued exposure.

Genome-wide epigenetic analyses have identified pollutant-sensitive loci whose methylation status correlates with both environmental exposure levels and cancer risk [97, 135]. Epigenome-wide association studies (EWAS) conducted in large cohorts pinpointed differentially methylated CpG sites that track ambient PM concentrations and predict increased incidence of lung cancer and other health outcomes [136]. Air pollution rewires the epigenome through coordinated changes in DNA methylation, histone modifications, and non-coding RNA profiles. This epigenetic reprogramming reshapes gene expression networks toward pro-tumorigenic states and may represent both a mechanism of disease initiation and a source of heritable risk signatures relevant for biomarker development.

### Inflammation and immune dysregulation

Chronic exposure to air pollutants initiates and sustains a pro-inflammatory cascade that plays a pivotal role in tumor initiation, promotion, and progression. Fine particulate matter (PM_2.5_), in particular, activates resident immune and epithelial cells in the lung, resulting in the release of a complex array of inflammatory mediators [137]. Upon inhalation, particle-laden alveolar macrophages and bronchial epithelial cells secrete pro-inflammatory cytokines and chemokines that recruit neutrophils, monocytes, and lymphocytes into the airways, creating a local inflammatory microenvironment in response to the oxidative stress [138].

Uptake of PM_2.5_ by bronchial epithelial cells and alveolar macrophages rapidly secrete IL-1β, IL-6, IL-8, and TNF-α [139]. These cytokines induces endoplasmic reticulum (ER) stress and activates mitogen-activated protein kinase (MAPK) pathways, culminating in the activation of transcriptional programs that upregulate genes linked to survival, proliferation, angiogenesis, and epithelial–mesenchymal transition (EMT), including NF-κB, AP-1, and JAK/STAT3 signaling cascades [140, 141]. Both in vivo animal models and in vitro human airway epithelial cultures exhibit substantial increases in IL-6 and TNF-α following PM exposure, which subsequently activate JAK/STAT signaling pathways [142, 143]. Specifically, phosphorylation of STAT3 promotes cell survival, proliferation, and angiogenesis, making it an indicator of inflammation-associated carcinogenesis [144]. NF-κB activation occurs via IκB degradation, allowing p65/p50 dimers to translocate to the nucleus and induce pro-survival genes such as BCL2 and COX-2 [145]. Simultaneously, AP-1 components (c-Fos, c-Jun) drive the transcription of IL-8, MMP-9, and VEGF, further increasing inflammation [146].

The JAK/STAT3 pathway plays a pivotal role in linking chronic inflammation to tumorigenesis. In PM_2.5_-exposed lung cells, autocrine IL-6 and TNF-α signaling activate STAT3 phosphorylation, promoting transcription of oncogenic and anti-apoptotic targets, like Cyclin D1, Survivin, and Mcl-1 [147]. Persistent STAT3 activity sustains a “feed-forward” loop, where inflammatory cytokines stimulate their own production and desensitize cells to apoptosis [148]. Similarly, exposure to the PAH component 1-nitropyrene provokes IL-1β, IL-6, and TNF-α secretion, along with keratinocyte chemoattractant, while concurrently activating PI3K/Akt signaling, which enhances cell survival [149].

Prolonged airway inflammation alters the immunologic and structural integrity of the tissue microenvironment and facilitates malignant developments. Alveolar macrophages, chronically burdened with particulate matter, exhibit an M2-like, tumor-associated phenotype characterized by defective phagocytosis, overproduction of IL-10, and secretion of matrix metalloproteinases [150]. These macrophages fail to clear transformed or apoptotic cells and instead promote angiogenesis and tissue remodeling, mimicking tumor-associated macrophages (TAMs) found in established cancers [151]. Chronic inflammation also impairs antitumor immunity, creating an ideal microenvironment for tumorigenesis [152]. Toll-like receptor 4 (TLR4) pathways and the NLRP3 inflammasome are aberrantly activated by pollutant components, leading to excessive IL-1β maturation and skewing alveolar macrophages towards a tumor-associated phenotype that supports immune evasion and tumor progression [153, 154].

Repeated exposure to IL-6 and TNF-α induces secondary DNA damage via nitric oxide (NO) and ROS released by infiltrating inflammatory cells [144]. Simultaneously, cytokine signaling supports epithelial–mesenchymal transition (EMT), a process by which epithelial cells lose polarity and cell–cell adhesion, acquiring mesenchymal traits that enhance cell motility and metastatic potential [155]. Persistent IL-6 and TNF-α signaling are particularly implicated in EMT and metastasis [156]. Chronic exposure induces transcriptional repressors such as Snail, Slug, and Twist, which suppress E-cadherin and promote a migratory, invasive phenotype [157]. These molecular changes also cause a morphological transition in epithelial cells from cobblestone-like to spindle-shaped, reflecting cytoskeletal reorganization and loss of cell–cell adhesion [158]. Prolonged PM_2.5_ exposure increases vimentin and N-cadherin expression, which are markers of EMT, through ROS-dependent activation of TGF-β/Smad and Wnt/β-catenin pathways [156].

Systemic immune dysregulation has also been observed in association with ambient PM_2.5_ exposure. Elevated levels of circulating inflammatory biomarkers, including C-reactive protein (CRP), soluble intercellular adhesion molecule-1 (sICAM-1), and soluble vascular cell adhesion molecule-1 (sVCAM-1), have been consistently reported in exposed populations, suggesting a state of low-grade, chronic systemic inflammation [159–161]. Alongside other pollutant-induced alterations, these inflammatory responses promote cellular transformation and tumorigenesis.

### Cell cycle dysregulation and apoptosis resistance

Chronic exposure to air pollutants disrupts cell cycle progression and programmed cell death, enabling the survival and proliferation of genomically unstable cells. Normally, genotoxic stress activates checkpoint pathways that halt the cell cycle and initiate DNA repair or apoptosis. Tumor suppressor p53 is central to this response, upregulating *CDKN1A* (encoding p21^WAF1/CIP1^), enforcing G_1_/S and G_2_/M arrest to prevent the propagation of damaged DNA [162].

However, persistent exposure to particulate pollutants such as diesel-derived PM_2.5_ can impair these checkpoint mechanisms and lead to unchecked proliferation. Promoter hypermethylation of TP53 and increased p53 ubiquitination and proteasomal degradation blunt its transcriptional activity, impairing the expression of target genes involved in DNA repair and cell cycle arrest [112]. Moreover, diesel PM_2.5_ exposure downregulates CDKN1A (p21^WAF1/CIP1^) via long noncoding RNA LINC00341–mediated post-transcriptional repression, permitting cell cycle progression at the G_2_/M checkpoint despite the presence of DNA lesions [104]. The combined suppression of p53 and p21 thus promotes unchecked cell-cycle advancement and accelerates the accumulation of mutations that drive neoplastic initiation.

Pollutants also reprogram apoptotic signaling pathways. Persistent activation of NF-κB signaling by pollutant-induced ROS and cytokines drives transcription of anti-apoptotic genes including BCL2, BCL-xL, and members of the inhibitor of apoptosis (IAP) family [163]. This pro-survival bias is reinforced by the sustained activation of the PI3K/AKT and MAPK pathways, which promote cell survival, metabolic adaptation, and resistance to death signals [164]. Long-term pollutant exposure has been shown to elevate Bcl-2/Bcl-xL protein levels while suppressing Bax, tipping the balance toward survival even in the presence of severe DNA damage [165]. In this state, damaged cells evade apoptosis, survive further mutagenic insults, and propagate oncogenic alterations.

PM_2.5_ exposure induces autophagy in lung epithelial cells through inhibition of the PI3K/Akt/mTOR pathway, allowing damaged cells to recycle organelles and resist apoptosis [166, 167]. Inhibition of autophagy restores apoptosis and cytotoxicity, confirming its protective role [168]. Long noncoding RNAs, like lncRNA H19, have also been implicated in pollutant-induced autophagy, modulates Beclin-1 expression to sustain autophagic flux under particulate stress [169]. Thus, chronic air pollution exposure fosters a cell population characterized by checkpoint failure, mitochondrial adaptation, and autophagy-dependent survival.

PM_2.5_ exposure is strongly associated with sustained cellular proliferation and malignant transformation. In pulmonary cell lines, treatment with PM_2.5_ increases proliferation, migration, and invasion, accompanied by transcriptomic signatures resembling invasive cancer phenotypes—marked by heightened motility and growth [170]. Mechanistically, IL-1β and MMP-1 are upregulated in response to PM_2.5_, serving as key mediators of its pro-proliferative effects. DNA damage responses also contribute: 3-nitrobenzanthrone (3-NBA), a potent nitro-PAH, induces phosphorylation of the ATM checkpoint kinase and S-phase accumulation in bronchial epithelial cells, along with p53-driven apoptosis following genotoxic stress [171]. Similarly, exposure to PAHs activates the aryl hydrocarbon receptor (AhR), promoting transcription of CYP1A1/1B1 and AhR-dependent proliferative signaling [172]. In vivo, adults chronically exposed to Mexico City air pollution exhibit a significant increase in S-phase nasal epithelial cells and sustained epithelial turnover within a week, suggesting persistent regenerative pressure [173]. Furthermore, PM_2.5_-treated lung cancer cells release exosomes enriched in Wnt3a, which activate β-catenin signaling in recipient cells and drive proliferation and tumor progression in a Wnt3a-dependent manner [174].

Angiogenesis, essential for tumor growth and metastasis, is strongly promoted by particulate matter exposure. PM_10_ induces dose-dependent microvessel formation and dense inflammatory infiltrates in the chick embryo chorioallantoic membrane assay detectable after 4 days of exposure [175]. PAHs, notably benzo[a]pyrene (BaP), activate pro-angiogenic signaling cascades by upregulating Akt kinase and HIF-1α in A549 and lung fibroblast cells, leading to enhanced VEGF expression [176]. Exposure to PM_2.5_ compromises endothelial barrier integrity by inducing cytoskeletal reorganization and reducing trans-endothelial electrical resistance, thereby promoting vascular permeability and leakage [177]. Both PAHs and transition metals within particulate matter further stimulate angiogenic mediators, like including VEGF, FGF, and IL-8, amplifying endothelial activation and neovessel formation [178]. Mice exposed to PM_2.5_ exhibit elevated serum concentrations of twelve angiogenesis-associated factors alongside increased tumor vascularization [179]. Further, PM_2.5_ exposure reshapes the methylome of BEAS-2B cells, deregulating genes involved in angiogenesis and endothelial remodeling [108]. Pollutant exposure promotes angiogenesis through inflammatory, oxidative, and epigenetic pathways, fostering a pro-tumorigenic microenvironment. Air pollution perturbs core cell cycle and apoptotic pathways through both transcriptional repression and post-translational modulation of key regulatory proteins. The resulting dysfunctional cellular response contributes to a permissive state for chronic environmental-induced carcinogenesis.

## Microplastics and emerging patterns in environmental carcinogenesis

Recent years have witnessed growing concern over the role of airborne microplastics (MPs), or ubiquitous synthetic polymer fragments under 5 mm in diameter, as emerging environmental carcinogens [180]. These synthetic polymer fragments are now recognized as components of ambient PM that may contribute to the carcinogenic potential of air pollution [181]. Airborne microplastic fibers and fragments have been detected in both indoor and outdoor air, where they can be inhaled and deposited deep in the respiratory tract [182, 183]. Unlike classical pollutants such as benzene or arsenic, MPs do not have established toxicological profiles and cause harm through a combination of mechanical, chemical, and immunological mechanisms [184]. Humans are exposed to microplastics during use of common plastic products, like packaging containers, synthetic textiles, and hygiene products. Exposure can also occur through direct ingestion, inhalation of airborne microplastic fibers and particles, or dermal contact [185]. These particles are now detected in virtually every environmental compartment—air, water, soil and increasingly, in human tissues, including lung, liver, kidney, placenta, and blood [186]. Notably, several studies have reported that tumor tissues (e.g., colorectal, lung, and bladder) harbor higher concentrations of MPs than adjacent healthy tissue, raising the possibility of selective accumulation and biologic relevance in cancer pathogenesis [187–189].

Microplastics exposure may contribute to changing incidence patterns in certain cancer types. Young-onset colorectal cancer (CRC) has risen dramatically in high-income countries, often without clear familial or dietary explanations [190]. Emerging studies now hypothesize that chronic ingestion of microplastics via processed food, drinking water, and environmental contamination may contribute to this trend by damaging barrier integrity of the colonic mucus layer, disrupting gut microbiota balance, and promoting mutagenic inflammation [191, 192]. Microplastic exposure may contribute to rising rates of hormone-sensitive cancers, such as breast and prostate cancer, in younger populations. Chronic low-dose exposure to endocrine-disrupting plasticizers disrupts hormonal signaling pathways, alters expression of cell-cycle transcripts, and fuels inflammatory responses in human breast tissue [180]. Increased exposure to microplastics and pollution has been proposed to help explain shifting age distributions and rising incidence of estrogen receptor-positive breast cancer and early-onset prostate cancer in some industrialized regions [193].

Recent studies confirm that microplastics (MPs) are present in many human tissues [186]. In one analysis of solid tumor biopsies, pyrolysis–GC/MS identified three MP polymers – polystyrene (PS), polyvinyl chloride (PVC), and polyethylene (PE) – in tumor tissue. Of 61 tumors (lung, stomach, colon, cervix, pancreas) tested, MPs were found in 26 samples, with high detection rates in lung (80%), gastric (40%), colon (50%), cervical (17%), and pancreatic (70%) tumors [188]. PS was the most common polymer (found in 20 samples, ~ 59.6 ng/g tissue), followed by PVC (17 samples, ~ 52.0 ng/g) and PE (11 samples, ~ 86.9 ng/g) [189]. Notably, lung cancers showed the highest MP incidence, quantity and diversity, suggesting a potential link between airborne microplastic exposure and pulmonary tumorigenesis [188]. Smoking further increases microplastic exposure through inhalation of microfibers and plastic-derived particles, which may contribute to idiopathic interstitial lung disease and lung adenocarcinoma [194, 195].

Beyond their established cytotoxic effects, emerging evidence suggests that MPs contribute to tumor progression by modulating the tumor microenvironment (TME). Studies have shown that MPs can interact with various cellular and extracellular components of the TME, including tumor cells, immune cells (e.g., macrophages), fibroblasts, endothelial cells, and the extracellular matrix [196]. The TME of pancreatic tumors containing MPs have a statistically significant reduction in CD8 + T cells (*p* = 0.0023), NK cells (*p* = 0.0224), and dendritic cells (*p* = 0.0052), as well as an increase in the number of neutrophils (*p* = 0.0144) [188], which may influence efficacy of immunotherapy. PS and PE particles have been shown to increase cytokine release, induce ROS production, and activate key inflammatory pathways such as NF-κB and NLRP3, thereby sustaining chronic inflammation and immune dysregulation [197]. MPs may also impair structural elements of the TME, weakening epithelial barriers and impacting cell-matrix adhesion [198].

Mechanistically, microplastics (MPs) act as physical irritants that can provoke chronic inflammation upon deposition in human tissues. Their morphological characteristics of the cubic, spherical, rod-shaped, and irregular fragments determine the extent of tissue irritation and injury. Irregularly shaped and sharp-edged particles are particularly harmful, as they can physically damage epithelial surfaces, simulate and injure mucous membranes, and obstruct parts of the digestive tract [184]. Ultrafine MPs (< 1 mm) are especially concerning because they can bypass primary tissue barriers, penetrate capillaries, and disperse systemically via the bloodstream, where their hydrophobic surfaces may disrupt cellular homeostasis. Compared to spherical particles, irregular fragments are more likely to cause mechanical irritation and have been linked to more severe respiratory symptoms, such as coughing and dyspnea [198]. Interpretation of microplastic toxicity remains complicated by the lack of standardized exposure mixtures and reference materials. Variations in polymer composition, particle morphology, and surface chemistry likely influence the extent of oxidative stress and inflammatory responses. However, current studies provide insufficient evidence to determine whether these physical characteristics or overall dose are the primary predictors of biological impact.

MPs also act as chemical irritants that exacerbate the physical inflammatory response. Their high surface-area-to-volume ratio makes them effective vectors for adsorbed pollutants, such as heavy metals, polycyclic aromatic hydrocarbons (PAHs), and phthalates, which themselves possess carcinogenic potential [199]. In vitro and animal models have shown that ingested or inhaled microplastics can generate oxidative stress, impair DNA repair, and activate inflammatory signaling, notably the MAPK and NF-κB pathways [200]. The resulting genomic instability and immune dysregulation constitute early hallmarks of carcinogenesis.

There are emerging concerns regarding the endocrine-disrupting potential of plasticizers such as bisphenol A (BPA) and phthalates, which leach from microplastic surfaces and have been implicated in hormone-sensitive cancers [201, 202]. Epidemiologic and laboratory studies link BPA exposure to increased risk of breast and prostate cancers through estrogen receptor activation and epigenetic alterations [201]. BPAs leached from microplastics can cross the blood-brain barrier, and exposure has been linked to alterations in the neuroendocrine system and signaling pathways, contributing to neuropsychological dysfunction, neurobehavioral disorders, and neurodegenerative diseases [201]. BPA exposure is linked to adverse effects in multiple tissues and organs, including the immune, reproductive, and neuroendocrine systems [203]. BPAs can cross the placental barriers and have been detected in fetal serum during gestation, suggesting that plastic exposure begins in utero and continues across the lifespan [204]. Researchers hypothesize that prenatal exposure to BPAs might impair neurodevelopment and may influence cancer risk windows across developmental and adult stages [205, 206].

Furthermore, recent research seeks to characterize the molecular signatures of microplastic-related cancers, which may include distinct mutational spectra, epigenetic modifications, or immune profiles that differentiate them from tumors driven by other environmental carcinogens [207]. Preliminary results show that tumors associated with high microplastic burden show upregulation of clathrin-mediated endocytosis machinery, may facilitate the accumulation of microplastics within the lysosome and activation of intracellular stress response [208, 209]. Microplastic-exposed epithelial cells exhibit altered expression of detoxification enzymes, like cytochrome P450s, and increased levels of metalloproteins and pro-inflammatory cytokines commonly implicated in tumor-initiation, promotion, and immune evasion, like IL-6 and TNF-α [211, 212]. Long-term exposure to MPs has also been associated with global DNA hypomethylation and histone modification patterns linked to genomic instability, suggesting that MPs actively reprogram epigenetic modifications and cellular pathways that drive malignant transformation [212].

## Conclusion

Air pollution remains a leading environmental cause of cancer, acting through complex mechanisms involving oxidative stress, DNA damage, epigenetic remodeling, inflammation, and immune dysregulation. While previous work has focused on characterizing particulate matter and chemical toxicants, emerging research suggests airborne microplastics may represent an understudied and biologically active component of the carcinogenic exposome. These synthetic particles act as physical and chemical irritants and also modulate key oncogenic pathways to reshape the tumor microenvironment. Though the field is still nascent, the detection of microplastics in human tumor tissue and their association with pro-carcinogenic molecular signatures highlight the need to better understand the role of microplastics in the broader carcinogenic exposome.

## Data Availability

No datasets were generated or analysed during the current study.

## Abbreviations

4-HNE: 4-hydroxynonenal
8-oxo-dG: 8-hydroxy-2′-deoxyguanosine
AhR: Aryl hydrocarbon receptor
Akt: Protein kinase B
AP: Abasic site
AP-1: Activator protein 1
As: Arsenic
ATP: Adenosine triphosphate
BaP: Benzo[a]pyrene
BER: Base excision repair
BPDE: Benzo[a]pyrene diol epoxide
Ca: Calcium
Cd: Cadmium
COX2: Cyclooxygenase-2
Cu: Copper
CYP: Cytochrome P450
DLEC1: Deleted in lung and esophageal cancer 1
DNA: Deoxyribonucleic acid
DNMT3B: DNA methyltransferase 3B
DSB: Double-strand break
EGFR: Epidermal growth factor receptor
EMT: Epithelial–mesenchymal transition
ER: Endoplasmic reticulum
ERK: Extracellular signal-regulated kinase
Fe: Iron
Fe^2+^/Fe^3+^: Ferrous / Ferric ion
GCLM: Glutamate–cysteine ligase modifier subunit
GSTM1: Glutathione S-transferase Mu 1
H2O2: Hydrogen peroxide
H3K4me3: Trimethylation of histone H3 at lysine 4
H3K9ac: Acetylation of histone H3 at lysine 9
H3K27ac: Acetylation of histone H3 at lysine 27
H4K16ac: Acetylation of histone H4 at lysine 16
HAT: Histone acetyltransferase
HDAC: Histone deacetylase
HNE: 4-hydroxynonenal (same as 4-HNE)
HO-1: Heme oxygenase 1
HR: Homologous recombination
IL: Interleukin
IL-1β: Interleukin-1 beta
IL-6: Interleukin-6
IL-8: Interleukin-8
IκB: Inhibitor of κB
IARC: International Agency for Research on Cancer
JAK: Janus kinase
JNK: c-Jun N-terminal kinase
Keap1: Kelch-like ECH-associated protein 1
LINE: 1-Long interspersed nuclear element-1
lncRNA: Long non-coding RNA
LOC146880: Long non-coding RNA LOC146880
MAPK: Mitogen-activated protein kinase
MDA: Malondialdehyde
MGMT: O-6-methylguanine-DNA methyltransferase
miRNA: MicroRNA
miR: MicroRNA (used with numbered designations)
Mn: Manganese
MP: Microplastic
MPs: Microplastics
mTOR: Mechanistic target of rapamycin
NER: Nucleotide excision repair
NEAT1: Nuclear paraspeckle assembly transcript 1 (lncRNA)
Ni: Nickel
NFκB: Nuclear factor kappa-light-chain-enhancer of activated B cells
NO_x_: Nitrogen oxides
NQO1: NAD(P)H dehydrogenase quinone 1
Nrf2: Nuclear factor erythroid 2—related factor 2
O_2_^−^: Superoxide anion
O3: Ozone
OGG1: 8-oxoguanine DNA glycosylase
PAH: Polycyclic aromatic hydrocarbon
PAHs: Polycyclic aromatic hydrocarbons
PARP: Poly(ADP-ribose) polymerase
p16: Cyclin–dependent kinase inhibitor 2 A (CDKN2A)
PIK3CA: Phosphatidylinositol-4,5-bisphosphate 3-kinase catalytic subunit alpha
PM: Particulate matter
PM_0.1_: Ultrafine particulate matter (< 0.1 μm)
PM_2.5_: Fine particulate matter (≤ 2.5 μm)
PM_10_: Coarse particulate matter (≤ 10 μm)
RAD51: DNA repair protein RAD51 homolog
RASSF1A: Ras association domain family member 1 A
RNA: Ribonucleic acid
ROS: Reactive oxygen species
SOD: Superoxide dismutase
SOX2: SRY-box transcription factor 2
STAT3: Signal transducer and activator of transcription 3
TAM: Tumor-associated macrophage
TNF-α: Tumor necrosis factor alpha
TP53: Tumor protein p53
TRAP: Traffic-related air pollution
TNS3: Tensin-3
VOC: Volatile organic compound
VOCs: Volatile organic compounds
Wnt: Wingless-related integration site signaling
